# Anti-Inflammatory and Antioxidant Components from *Hygroryza aristata*

**DOI:** 10.3390/molecules16031917

**Published:** 2011-02-25

**Authors:** Yu-Ming Chung, Yu-Hsuan Lan, Tsong-Long Hwang, Yann-Lii Leu

**Affiliations:** 1 Graduate Institute of Natural Products, College of Medicine, Chang Gung University, Taoyuan 33302, Taiwan; E-Mails: u97831001@cc.kmu.edu.tw (Y.-M.C.); htl@mail.cgu.edu.tw (T.-L.H.); 2 School of Pharmacy, China Medical University, Taichung 40402, Taiwan; E-Mail: lanyh@mail.cmu.edu.tw (Y.-H.L.)

**Keywords:** *Hygroryza aristata*, Gramineae, hygarine, anti-inflammatory, antioxidant

## Abstract

Twenty-six known compounds and two new compounds, including a new lignan, (7S*,8R*,7’R*,8’S*)-icariol A_2_-9-*O*-β-xylopyranoside (**1**), and a new indole alkaloid, hygarine (**2**), were isolated from the extracts of *Hygroryza aristata* (Gramineae). The structures of all compounds were elucidated on the basis of NMR spectral analysis. The compounds (-)-epigallocatechin-3-*O*-gallate (**4**) and (-)-epicatechin-3-*O*-gallate (**5**) possess free radical scavenging activities and compound **1** could inhibit superoxide anion generation and elastase release by fMLP/CB-induced human neutrophils with IC50 values of 19.33 ± 0.86 and 24.14 ± 1.59 μM, respectively.

## 1. Introduction

**Figure 1:**
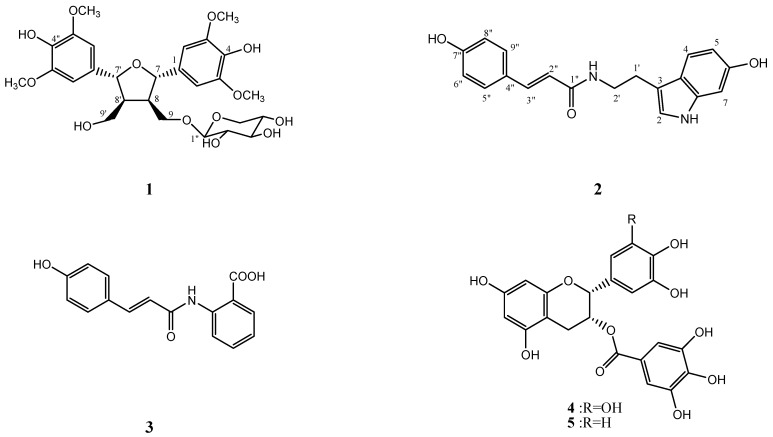
Structures of compounds **1–5**.

*Hygroryza aristata* (Gramineae) is a stoloniferous perennial plant widely distributed in tropical Asia [1]. This plant grows in paddy fields and ponds at 400–800 meters above sea level, often forming floating clusters in lakes and slow-flowing rivers, and is a forage favored by cattle. Nevertheless, to our knowledge the chemical and biological activities of this plant have not been reported previously. In the search for biologically active constituents from natural sources, we determined that the methanolic extract of this plant was able to scavenge DPPH free radicals. In the present study two new compounds, (7S*,8R*,7’R*,8’S*)-icariol A_2_-9-*O*-β-xylopyranoside (**1**) and hygarine (**2**), were isolated together with 26 known compounds from the extracts of the *H. aristata* (Figure 1). Several of these compounds were evaluated for their antioxidant and anti-inflammatory activities.

## 2. Results and Discussion

Compound **1** was obtained as a colorless syrup. Its IR spectrum exhibited hydroxyl and aromatic group absorption bands at 3,375 cm^−1^ and 1,517 cm^−1^, respectively. The UV spectrum revealed absorption maxima at 237 and 279 nm. The ^1^H-NMR data (Table 1) showed two typical 1,3,4,5-tetrasubstituted phenyl ring protons at δ 6.75 and 6.79 (each 2H, *s*), one anomeric proton at δ 4.23 (1H, *d*, *J* = 7.6 Hz) and four methoxyl groups at δ 3.87 (12H, s). Four methines at δ 2.34, 2.46, 5.01, 5.10 and two methylenes at δ 3.62, 3.67, 3.74, 4.01 were observed in the ^1^H-NMR and HMQC spectra. The ^13^C signals at δ 66.0, 70.2, 73.9, 77.0, 104.3 and the coupling constant (*J* = 7.6 Hz) of anomeric proton suggested that **1** contained a β-d-xylose moiety. 

**Figure 2:**
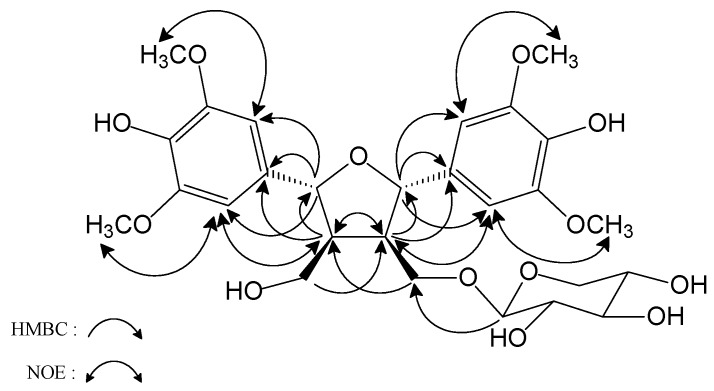
HMBC and NOE Correlations of compound **1**.

HMBC correlations between H-1’, H-2’, H-6’/C-7’ and H-1, H-2, H-6/C-7 indicated that the 1,3,4,5- tetrasubstituted phenyl rings were connected to C-7’ and C-7, respectively (Figure 2). The ^3^*J*-correlation signals between H-9 (δ_H_ 3.62, 4.01) and C-1” (δ_C_ 104.3) indicated the presence of a xylose unit attached at C-9. 

The planar structure of **1** is similar to that of icariol A_2_-9-*O*-β-xylopyranoside [2,3]. In a NOESY experiment, H-8/ H-2, 6; H-8’/H-2’, 6’ and H-8/H-8’ NOE correlations were observed, revealing that H-8, 8’ and the two phenyls are a *syn* conformation. Based on these results, we established the relative configuration of **1** as shown in Figure 2. Thus, the structure of compound **1** was determined to be (7S*,8R*,7’R*,8’S*)-icariol A_2_-9-*O*-β-xylopyranoside.

Compound **2** was obtained as a brown syrup with an elemental composition of C_19_H_18_N_2_O_3_, as determined by its HRFAB-MS ([M+1]^+^ *m/z* 323.3734). The IR spectrum of **2** displayed absorption bands from a hydroxyl group (3,404 cm^−1^) and an amide group (1,643 cm^−1^). In the UV spectrum of **2**, absorption maxima were observed at 224 (sh), 288, and 312 (sh) nm, revealing that the compound has an indole skeleton [4]. 

**Figure 3:**
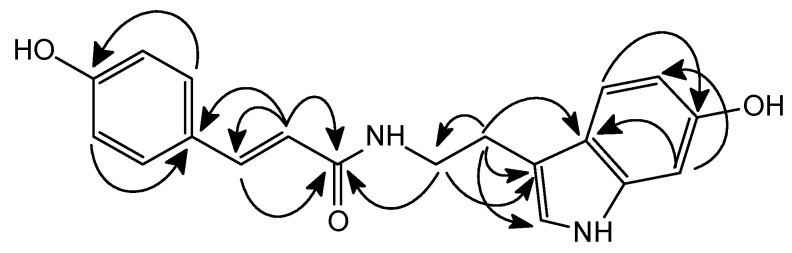
Significant correlations observed in HMBC of compound **2**.

In the ^1^H-NMR data of **2**, shown in Table 2, the three mutually coupled aromatic protons at δ 6.70 (1H, *dd*, *J* = 8.4, 2.0 Hz ), 7.03 (1H, *d*, *J* = 2.0 Hz ) and 7.19 (1H, *d*, *J* = 8.4 Hz) were assigned to H-5, H-7 and H-4 in the indole skeleton, respectively. The signal at δ 7.09 (1H, d, *J* = 2.4 Hz) was contributed by H-2 in the indole skeleton. In the aromatic region of the ^1^H-NMR spectrum, the signals at δ 6.85 (2H, *d*, *J* = 8.4 Hz) and 7.42 (2H, *d*, *J* = 8.4Hz) indicated the presence of a 1,4-disubstituted benzene ring, and *trans*-olefinic protons appeared at δ 6.49 (1H, *d*, *J* = 16.0 Hz) and 7.48 (1H, *d*, *J* = 16.0 Hz). Therefore, the combined data for compound **2** indicated the presence of a partial coumaric acid. Two methylene proton signals at δ 2.89 (2H, *t*, *J* = 7.6 Hz) and 3.59 (2H, *dd*, *J* = 13.6, 7.6 Hz) correlated with the carbon signal at δ 26.1 (C-1’) and 40.2 (C-2’) in the HSQC experiment. The COSY spectrum of **2** showed two methylenes coupled with NH (δ_H_ 7.30). Thus, compound **2** contains a partial NH-CH_2_-CH_2_ structure. The HMBC spectrum exhibited correlations between H-1’/C-2, C-3 and C-9, H-2’/C-1” and C-1’, H-2”/C-1”, C-3”, and C-4” indocatimg the three aforementioned structures to be connected as shown in Figure 3. In addition, the presence of a carbonyl group in the coumaric acid moiety connected to NH with an amide bond was further confirmed by the fragment at *m*/*z* 147 in the FAB-MS spectrum. According to previous reports and the above NMR analysis, compound **2** was deduced to have the structure shown and it was named hygarine.

Twenty-six known compounds, including *N*-*p*-coumarylanthranilic acid (**3**) [5], (-)-epigallo- catechin-3-*O*-gallate (**4**) [6], (-)-epicatechin-3-*O*-gallate (**5**) [7], L-tyrosine (**6**) [8], quercetin (**7**) [9], rutin (**8**) [9], kaempferol (**9**) [10], adenine (**10**) [11], adenosine (**11**) [11], inosine (**12**) [11], uracil (**13**) [12], uridine (**14**) [13], coumaric acid (**15**) [14], methyl coumarate (**16**) [15], ferulic acid (**17**) [16], *n-*hexacosyl ferulate (**18**) [17], vanillic acid (**19**) [18], *p*-hydroxybenzoic acid (**20**) [19], 2,6-di- methoxyquinone (**21**) [20], physcion (**22**) [21], cycloeucalenol (**23**) [22], squalane (**24**) [23], a *β*-sitosterol and stigmasterol mixture (**25**) [15], eicosanoic acid 2,3-dihydroxypropyl ester (**26**) [24], butcosanoic acid 2,3-dihydroxypropyl ester (**27**) [24] and sucrose (**28**) [25], were isolated and identified by comprehensive studies of their physical and spectral data. 

**Figure 4:**
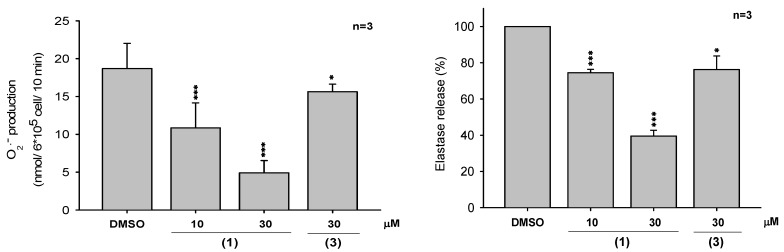
Effect of (7S*,8R*,7’R*,8’S*)-icariol A_2_-9-*O*-β-xylopyranoside (**1**) and *N*-*p*-coumarylanthranilic acid (**3**) isolated from *H. aristata* on generation of superoxide anion (left) and elastase release (right) in fMLP/CB-activated human neutrophils.

**Figure 5:**
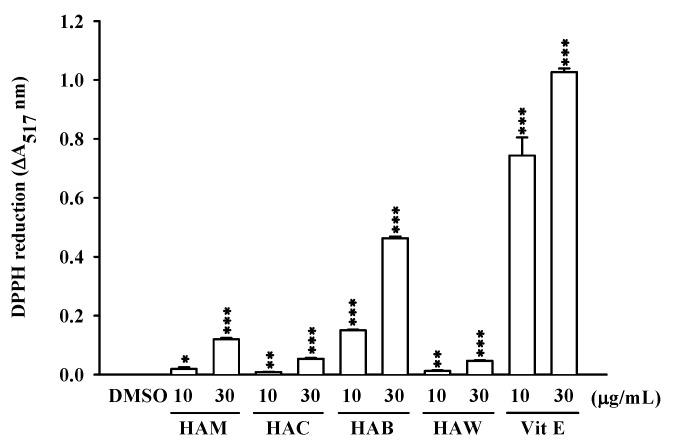
Effects of different crude extracts in scavenging DPPH radicals.

**Figure 6:**
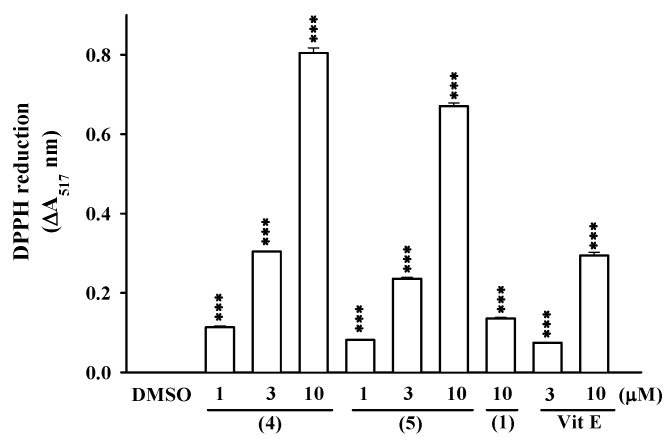
DPPH radical scavenging effects of compounds **1**, **4** and **5** isolated from the *n*-butanol layer.

Among all these compounds, several were evaluated for their antioxidant and anti-inflammatory activities. The inhibitory activity of (7S*,8R*,7’R*,8’S*)-icariol A_2_-9-*O*-β-xylopyranoside (**1**) and *N*-*p*-coumarylanthranilic acid (**3**) against the inflammatory response in human neutrophils was investigated. Compound **1** showed moderate anti-inflammatory activities and further, it inhibited superoxide anion generation and elastase release by fMLP/CB-induced human neutrophils, with IC50 values of 19.33 ± 0.86 and 24.14 ± 1.59 μM, respectively (Figure 4). In addition, the *n*-butanol soluble layer of the *H. aristata* methanolic extract was found to be a powerful scavenger of DPPH free radicals (Figure 5). In further bioactivity-guided investigation of the butanol layer, (-)-epigallo- catechin-3-*O*-gallate (**4**) (2.92 g, 0.31% of plant material), and (-)-epicatechin-3-*O*-gallate (**5**) (0.55 g, 0.06%) were obtained; these compounds were the major components of *H. aristata*. Their radical scavenging activities were greater by approximately 3.8 and 3.1-fold, respectively, than that of α-tocopherol (Figure 6). These results suggest that *H. aristata* could be a rich natural source of catechins due to its high content of these substances.

## 3. Experimental 

### 3.1. General 

Melting points were measured on a Yanagimoto MP-S3 micro melting point apparatus and are uncorrected. The UV spectra were recorded on a Hitachi U-3010 spectrophotometer in MeOH solution. The IR spectra were recorded on a Jasco IR Report-100 spectrophotometer as KBr discs. The ^1^H- and ^13^C-NMR spectra were recorded on Bruker Avance-400 spectrometer. Chemical shifts are shown in δ values with tetramethylsilane as internal reference. The mass spectra were performed in the EI or FAB (matrix: glycerol) mode on a VG 70-250 S spectrometer. Specific rotations were determined on a Jasco P-1010 polarimeter. 

### 3.2. Plant Material 

*Hygroryza aristata* was collected and authenticated by Prof. C. S. Kouh at I-Lang, Taiwan. A voucher specimen (CGU-HA-1) was deposited in the herbarium of Chang Gung University, Taoyuan, Taiwan.

### 3.3. Extraction and Isolation 

Dry *H. aristata* (948 g) was extracted with MeOH (6 L × 6) under reflux for 8 hours and concentrated to give a brown syrup (122.0 g). The syrup was suspended in H_2_O and partitioned successively with CHCl_3_ and *n*-BuOH. The CHCl_3_ extract (17.0 g) was subjected to column chromatography over silica gel and eluted with a CHCl_3_ and MeOH step gradients to afford eight fractions. Repeated column chromatography of the first fraction, over silica gel with *n*-hexane and EtOAc mixtures yielded squalane (**24**, 2.3 mg). The second and third fractions were treated as the first fraction to obtain *n-*hexacosyl ferulate (**18**, 7.2 mg), *p*-hydroxybenzoic acid (**20**, 5.0 mg), 2,6-di- methoxyquinone (**21**, 2.2 mg) and physcion (**22**, 1.5 mg), separately. The fourth fraction was repeatedly chromatographed over silica gel with CHCl_3_ and MeOH (18:1) to afford cycloeucalenol (**23**, 7.1 mg), *β*-sitosterol & stigmasterol mixture (**25**, 100.0 mg), eicosanoic acid 2,3-dihydroxypropyl ester (**26**, 5.2 mg) and butcosanoic acid 2,3-dihydroxypropyl ester (**27**, 12.1 mg), successively. The fifth fraction was repeatedly column chromatographed over silica gel with CHCl_3_-MeOH gradients to give coumaric acid (**15**, 11.5 mg) and methyl coumarate (**16**, 5.3 mg). The sixth fraction was purified by recrystallisation to afford vanillic acid (**19**, 2.5 mg). 

The *n*-BuOH layer (18.4 g) was applied on Diaion HP-20 gel and eluted with gradients of H_2_O and MeOH to give eight fractions. The second fraction was subjected to column chromatography over silica gel and eluted with a CHCl_3_ and MeOH step gradient to afford L-tyrosine (**6**, 10.0 mg), inosine (**12**, 11.0 mg) and uridine (**14**, 20.0 mg), successively. The third fraction was chromatographed on silica gel column and eluted with CHCl_3_ and MeOH mixtures to give adenine (**10**, 15.6 mg) and uracil (**13**, 5.6 mg). The fourth fraction was filtered to give adenosine (**11**, 20.3 mg).The fifth fraction was repeatedly column chromatographed over silica gel with CHCl_3_ -MeOH gradients to give quercetin (**5**, 3.5 mg), kaempferol (**7**, 10.0 mg), (-)-epigallocatechin-3-*O*-gallate (**4**, 2,919.3 mg) and (-)-epi- catechin-3-*O*-gallate (**5**, 552.0 mg). The seventh fraction was also rechromatographed over silica gel and eluted with EtOAc to give (7S*,8R*,7’R*,8’S*)-icariol A_2_-9-*O*-β-xylopyranoside (**1**, 15.7 mg) and rutin (**6**, 3.2 mg). The eighth fraction was repeatedly column chromatographed over silica gel with CHCl_3_-MeOH gradients to give hygarine (**2**, 3.0 mg), *N*-*p*-coumarylanthranilic acid (**3**, 8.6 mg) and ferulic acid (**17**, 2.8 mg), separately.

The H_2_O layer (82.1 g) was applied on Diaion HP-20 gel and eluted with gradients of H_2_O and MeOH to give four fractions. The first fraction was chromatographed on Sephadex LH-20 column and eluted with gradients of H_2_O and MeOH to afford sucrose (**28**, 20.5 mg). The second fraction was repeatedly column chromatographed over Sephadex LH-20 with H_2_O:MeOH gradients to give uridine (**14**, 20.0 mg). 

### 3.4. DPPH Free-Radical Scavenging Activity

The scavenging activity of the DPPH radical was assayed by the modified method of Shimada *et al.* [26]. The absorbance was measured at 517 nm. Lower absorbance of the reaction mixture indicates higher free-radical scavenging activity. The DPPH radicalscavengingactivity (%) was calculated by the following equation:

scavenging activity (%) = (1 − *A*_sample_/*A*_control_) × 100%

### 3.5. Preparation of Human Neutrophils

Human neutrophils from venous blood of healthy, adult volunteers (20–30 years old) were isolated with a standard method of Dextran sedimentation prior to centrifugation in Ficoll Hypaque gradient and hypotonic lysis of erythrocytes. Purified neutrophils that contained > 98% viable cells, as determined by trypan blue exclusion, were resuspended in HBSS buffer at pH 7.4, and kept at 4 °C before use.

### 3.6. Measurement of Superoxide Anion (O_2_^•−^) Generation

The measurement of the generation of O_2_^•−^ was based on the superoxide dismutase (SOD)-inhibitable reduction of ferricytochrome *c* [27]. In brief, after supplementing with ferricytochrome *c* (0.5 mg/mL), neutrophils (6 × 10^5^/mL) were equilibrated at 37 °C for 2 min and incubated with either control or different concentrations of tested compounds for 5 min. Cells were activated by fMLP (0.1 μM) or PMA (5 nM) for 10 min. When fMLP was used as stimulant, cytochalasin B (CB, 1 μg/mL) was incubated for 3 min before peptide activation. The changes in absorbance with the reduction of ferricytochrome *c* at 550 nm were continuously monitored in a double-beam, six-cell positioner spectrophotometer with constant stirring. Calculation is based on the difference of the reactions with and without SOD (100 U/mL) divided by the extinction coefficient for the reduction of ferricytochrome *c* (ε = 21.1/mM/10 mm).

(7S*,8R*,7’R*,8’S*)-Icariol A_2_-9-O-β-xylopyranoside (**1**). Colorless syrup. Formula: C_27_H_36_O_13_. [α]_D_ +0.63° (*c* 0.35, MeOH). UV λ_max_nm (logε) : 237 (2.89), 279 (2.70). IRν_max_ cm^−1^: 3,375, 1,613, 1,517, 1,113. FAB-MS *m/z* (*rel. int.%*): 591 ( [M+Na]^+^, 8), 175 (7), 161 (9), 153 (6), 149 (20), 133 (9). ^1^H- and ^13^C-NMR: see Table 1.

*Hygarine* (**2**). Brown syrup. UV λ_max_nm (logε): 224 (sh) (3.52), 288 (3.27), 312 (sh) (3.22). IRν_max_ cm^−1^: 3,404, 1,643, 1,048. FAB-MS *m/z* (*rel. int.%*): 323 ([M+1]^+^, 12), 147 (30), 55 (100), 94 (30). HRFAB-MS: Anal. Calcd. for C_19_H_18_N_2_O_3_ 323.3726, found 323.3734. ^1^H- and ^13^C-NMR: see Table 2.

## 4. Conclusions

Two new compounds, (7S*,8R*,7’R*,8’S*)-icariol A_2_-9-*O*-β-xylopyranoside (**1**) and hygarine (**2**), together with twenty-six known compounds, were isolated from the extracts of *Hygroryza aristata*. (-)-Epigallocatechin-3-*O*-gallate (**4**) and (-)-epicatechin-3-*O*-gallate (**5**) possess free radical scavenging activities. (7S*,8R*,7’R*,8’S*)-Icariol A_2_-9-*O*-β-xylopyranoside (**1**) could inhibit superoxide anion generation and elastase release by fMLP/CB-induced human neutrophils with IC50 values of 19.33 ± 0.86 and 24.14 ± 1.59 μM, respectively. 

## Figures and Tables

All data are expressed as mean ± S.E.M. (n = 3). *P < 0.05, ***P < 0.001 compared with the control value.

Reduction of DPPH was measured spectophotometrically at 517 nm. All data are expressed as the mean ± S.E.M. of three separate experiments. *P < 0.05, **P < 0.01, ***P < 0.001 compared with the control value. HAM: *H. aristata* methanolic crude extract, HAC: *H. aristata* CHCl_3_ layer, HAB: *H. aristata n*-BuOH layer, HAW: *H. aristata* water layer, Vit E: α-tocopherol.

Reduction of DPPH was measured spectophotometrically at 517 nm. All data are expressed as the mean ± S.E.M. of three separate experiments. ***P < 0.001 compared with the control value.

**Table 1:** ^1^H- and ^13^C-NMR data for compound **1** (400 and 100 MHz in methanol-*d*_4_).

Position	δ_C_	δ_H_
1	133.2	
2, 6	103.9	6.75 (2H, *s*)
3, 5	148.3	
4	135.2	
7	83.6	5.10 (1H, *d*, *J* = 8.4 Hz)
8	50.9	2.34 (1H, *m*)
9a 9b	68.6	3.62 (1H, *m*) 4.01 (1H, *dd*, *J* = 10.0, 4.8 Hz)
1’	133.2	
2’, 6’	104.0	6.79 (2H, *s*)
3’, 5’	148.4	
4’	135.3	
7’	83.3	5.01 (1H, *d*, *J* = 8.4 Hz)
8’	53.5	2.46 (1H, *m*)
9’a 9’b	60.1	3.67 (1H, *m*) 3.74 (1H, *dd*, *J* = 11.6, 4.4 Hz)
3, 5, 3’, 5’-OCH_3_	55.9	3.87 (12H, *s*)
Xyl-1	104.3	4.23 (1H, *d*, *J* = 7.6 Hz)
Xyl-2	73.9	3.20 (1H, *m*)
Xyl-3	77.0	3.31 (1H, *m*)
Xyl-4	70.2	3.48 (1H, *m*)
Xyl-5	66.0	3.20 (1H, *m*) 3.84 (1H, *dd*, *J* = 6.4, 2.4 Hz)

**Table 2:** ^1^H- and ^13C^-NMR data for compound **2** (400 and 100 MHz in acetone-*d*_6_).

Position	δ_C_	δ_H_
2	123.5	7.09 (1H, *d*, *J* = 2.4 Hz)
3	112.1	
4	112.0	7.19 (1H, *d*, *J* = 8.4 Hz)
5	111.9	6.70 (1H, *dd*, *J* = 8.4, 2.0 Hz)
6	151.1	
7	103.0	7.03 (1H, *d*, *J* = 2.0 Hz)
8	132.0	
9	128.8	
1’	26.1	2.89 (2H, *t*, *J* = 7.6 Hz )
2’	40.2	3.59 (2H, *dd*, *J* = 13.6, 7.6 Hz)
1”	166.1	
2”	119.4	7.48 (1H, *d*, *J* = 16.0 Hz)
3”	139.6	6.49 (1H, *d*, *J* = 16.0 Hz)
4”	127.3	
5”, 9”	129.6	7.42 (2H, *d*, *J* = 8.4 Hz)
6”, 8”	116.1	6.85 (2H, *d*, *J* = 8.4 Hz)
7”	159.3	
1-NH		7.74 (1H, br*.s*)
3’-NH		7.30 (1H, br.*s*)
6-OH		8.86 (1H, br.*s*)
7”-OH		9.75 (1H, br.*s*)
